# Opportunities and challenges in providing health care for International Retirement Migrants: a qualitative case study of Canadians travelling to Yuma, Arizona

**DOI:** 10.1186/s40794-020-00110-6

**Published:** 2020-06-03

**Authors:** John Pickering, Valorie A. Crooks, Jeremy Snyder, Trudie Milner

**Affiliations:** 1 Simon Fraser University, Burnaby, Canada; 2 Yuma Regional Medical Center, Yuma, Arizona USA

**Keywords:** International retirement migration, Canada, United States, Arizona, Health care, Hospital

## Abstract

**Background:**

Increasing numbers of older individuals opt to spend extended time abroad each year for lifestyle, health, and financial reasons. This practice is known as international retirement migration, and it is particularly popular among retirees in Global North countries such as Canada. Despite the popularity of international retirement migration, very little is known about how and why health care is accessed while abroad, nor the opportunities and challenges posed for destination hospitals. In this article we focus on addressing the latter knowledge gap.

**Methods:**

This qualitative case study is focused on the only hospital in Yuma, Arizona – a popular destination for Canadian retirement migrants in the United States. We conducted focus groups with workers at this hospital to explore their experiences of treating this transnational patient group. Twenty-seven people participated in three, 90-min focus groups: twelve nurses, six physicians, and nine administrators. Thematic analysis of the focus group transcripts was conducted using a triangulated approach.

**Results:**

Participants identified three care environments: practice, transnational, and community. Each environment presents specific opportunities and challenges pertaining to treating Canadian retirement migrants. Important opportunities include the creation of a strong and diverse seasonal workforce in the hospital, new transnational paths of communication and information sharing for physicians and health administrators, and informal care networks that support formal health care services within and beyond the hospital. These opportunities are balanced out by billing, practical, administrative, and lifestyle-related challenges which add complexity to treating this group of transnational patients.

**Conclusion:**

Canadians represent a significant group of patients treated in Yuma, Arizona. This is contrary to long-standing, existing research that depicts older Canadians as being reluctant to access care while in the United States. Significant overlaps exist between the opportunities and challenges in the practice, transnational and community environments. More research is needed to better understand if these findings are similar to other destinations popular with Canadian international retirement migrants or if they are unique to Yuma, Arizona.

## Background

International retirement migration is a voluntary residential strategy [1] that occurs when older people around the age of retirement or thereafter – and typically living in Global North countries – relocate abroad [2, 3]. This relocation can happen on a short-term or permanent basis. In this article we are focused on short-term international retirement migration by older Canadians to the United States (US). Short-term international retirement migrants, or ‘snowbirds’ as they are sometimes popularly referred to in North America, are typically attracted to the warm, dry environments offered by popular destinations during what corresponds with the winter months in their home country. Retired Canadians are among those who take part in such international retirement migration. Estimates range from around 500,000 to more than one million Canadian snowbirds annually traveling to the US during the winter [4, 5], with some sources suggesting that these numbers grow by 2–3% annually [6, 7]*.* These are likely to be underestimates due to the lack of population-level quantifiable data and governmental oversight in both in- and out-migratory patterns of international retirement migrants [2, 3]. Previous research indicates the influx of older Canadian retirement migrants in the US is primarily to popular destinations in the southern states (e.g., Florida, Texas, Arizona, California). This seasonal migration of older Canadians can have many benefits for local economies, and recently proposed residency laws in the US are aimed at allowing Canadians to stay longer than 6 months and contribute more to the local economy [8]. While this increased seasonal demand is most clearly beneficial to the goods and service sectors and commercial retail outlets of destination economies, it is also felt in allied sectors such as real estate and health care [4, 9].

It is widely acknowledged that most international retirement migrants are in their sixties and beyond [10, 11]. This age group also corresponds with the stage in the life-course when people are likely to be managing one or more chronic health conditions (e.g., diabetes, arthritis), have a cancer diagnosis (or multiple diagnoses), and/or experience acute health episodes (e.g., heart attack, stroke) [12–14]. It is thus likely that at least some international retirement migrants will need to access health care abroad to manage chronic and/or acute episodes, in addition to treating injuries. Meanwhile, only a few studies have set out to explore how international retirement migrants manage their health while abroad by accessing health care in destination communities [1, 15–18]. The financial accessibility of health care in destinations is also widely cited as a factor that pulls international retirement migrants to particular places [19, 20]; though, again, research has not followed through with examining the lived experience of such access.

Although Canadian citizens and permanent residents have access to necessary health care with no out-of-pocket payment at the point of care, as provided under the Canada Health Act, this access does not extend to when they need to access care while travelling abroad [21]. Older Canadians who wish to have health insurance coverage while abroad, including in the US, do so by purchasing private travel health insurance policies. Existing research suggests that older Canadian international retirement migrants perceive the cost of health care in the US to be high and report they may prefer to take measures to avoid accessing care while abroad [9, 15]. This includes behaviours such as filling prescriptions before departure and/or seeing regular care providers upon return home. While this limited existing research is useful for understanding some important aspects of the experiences of older Canadian retirement migrants accessing health care while in the US, these dated - yet influential - studies offer no insight into this issue from the perspectives of health care providers. We address this knowledge gap in the current analysis.

In this exploratory qualitative study, we examine the challenges and opportunities surrounding Canadian retirement migrants’ use of and access to hospital-based health care while in the US from a unique perspective: that of health care providers and administrators in the destination. Specifically, we report on the results of focus groups conducted with workers at a regional medical centre in the Southern Arizona city of Yuma. Yuma was selected for this case study due to its popularity as a destination for Canadian snowbirds and also because the city has only one hospital and there are no nearby cities, which means that workers at this facility have regular contact with older Canadian retirement migrants. We conducted focus groups with three groups of workers at this hospital. Using thematic analysis of the focus group results, we explore the opportunities and challenges presented by providing hospital-based care to this mobile, transnational, and aging patient group. We analytically organize these opportunities and challenges according to the unique environments within which they emerge. We use the term ‘environments’ to refer to important, yet distinct, domains of engagement encountered throughout the course of Canadian retirement migrants being treated in the hospital setting as in- or out-patients while living seasonally in Yuma, Arizona.

## Methods

The purpose of this qualitative case study was to capture the experiential insights of destination-based health care providers delivering care to older Canadians while abroad in the context of international retirement migration. To address this purpose, we conducted focus groups with three health care provider groups working at a specific hospital: nurses, physicians, and administrators. Focus groups are an effective qualitative tool that can produce rich data through both the sharing of experiential insights by individual participants and the interaction between participants [22]. We specifically sought to generate new ideas through an organic group discussion, which made focus groups and appropriate choice over interviews [23, 24].

All participants were working at the sole hospital facility in Yuma, Arizona at the time of the study. This hospital has just over 400 beds, 2200 staff, and in recent years has seen approximately 1400 Canadian patient visits or admissions per year. Yuma is located in Southern Arizona, adjacent to the US-Mexico border. With no other hospitals within reasonable driving distance, it is likely that the Yuma Regional Medical Centre sees most (if not all) of the Canadian retirement migrants in the area in need of medical care. The city has a population of just over 100,000 and receives a sizeable number of Canadian retirees who travel abroad for extended stays each winter, with estimates of 80,000 to 100,000 additional residents during these months [25]. For these reasons, it was selected for our case study. Consistent with case study methodology [26, 27], we drew on multiple sources of information to understand important contextual aspects of Yuma as a destination for older Canadians. This included taking tours of health and social care sites in the city, reviewing publicly-available information in online discussion boards and websites about Yuma as a destination community for Canadians, and observing facets of everyday life in Yuma more broadly during fieldwork (including infrastructure targeting seniors). This wider contextual information informed both data collection and analysis.

### Recruitment

We sought to recruit up to 12 participants for each of three focus groups: one held with nurses, one with physicians, and one with administrators (e.g., directors, billing and staff coordinators, insurance liaisons). Our goal was to have 8–10 people participate in each focus group, which is consistent with the established norm that 6 to 12 participants per group is ideal in order to facilitate conversation and interaction while remaining manageable [22, 23]. We recruited up to 12 participants per group to accommodate for some people not attending on the day. Following approval for this study from Simon Fraser University’s Office of Research Ethics, our on-site collaborator circulated an invitation to participate to people in relevant departments at the hospital. The e-mail invitation contained information about the study purpose, proposed focus group dates, contact information for the lead investigators, and details on how to express interest in participating. It was also explained the focus group would be conversational in nature and thus participants need not have specific types of expertise with or established viewpoints on treating Canadian patients. Given the significant number of Canadian retirement migrants who seek treatment at the hospital each year we were confident all participants would have adequate knowledge of this patient group. Those interested in participating were asked to follow-up with the lead investigator by e-mail to confirm eligibility (i.e., they were a staff member at the hospital in one of the three target groups) and sign up to participate. This strategy also ensured our on-site collaborator would not know the identities of who did (or did not) participate in the focus groups in order to maintain anonymity. In keeping with current approaches to reporting on qualitative research, we do not include participant details with quotes [24, 28]. This assists with minimizing the risk of identification associated with all participants working for the same employer and the lack of other medical centres in the region.

### Data collection

All focus groups were held on weekdays in May, 2017 and were scheduled to run for 1.5 h each. Two were held in the morning, where we provided breakfast, and the third was held in the early evening, where we provided a light dinner. All focus groups were conducted in meeting rooms at the hospital, to least inconvenience the participants. Two investigators attended each focus group, taking turns between serving as moderator and note taker.

Following review and completion of consent forms, the focus groups began with a round of introductions as well as an overview of the study purpose. From there the conversation was guided by five broad questions that were developed by our investigative team following extensive review of the literature and multiple conversations with our on-site collaborator. These broad questions probed: familiarity with treating Canadian international retirement migrants and the types of care they often seek; impacts of the seasonal ‘snowbird’ population on health services locally and in the hospital specifically; understandings of why Canadian patients may opt for care in one facility over another while abroad; the practical realities of treating seasonal Canadian international retirement migrants; and ways to facilitate continuity of care in the context of transnational care provision. Most of the broad questions had sub-probes to stimulate discussion. After each focus group the note taker and moderator met with the on-site collaborator to have a debriefing conversation (identifying important issues emerging from the conversation, determining if changes needed to be made to the guide in subsequent focus groups, etc.).

### Analysis

Two digital recorders were used to record the discussion in each focus group (with one serving as a back-up). Following completion of the focus groups, the recordings were transcribed verbatim. Transcripts were independently reviewed by all investigators in preparation for coding and thematic analysis. Thematic analysis is a systematic, qualitative data analysis analytic technique in which researchers identify meaningful patterns that constitute themes amongst the collected data [29, 30]. Following independent transcript review, a meeting was held to identify emergent themes through contrasting specific issues discussed by participants against the existing literature and the contextual insights gathered for this qualitative case study through our on-site fieldwork. Through this process we identified three ‘environments’ offering distinct opportunities and challenges for treating Canadian international retirement migrant patients in the hospital setting as being a meaningful analytic focus. These environments serve as the focus of the current analysis. We use the term environments to refer to important, yet distinct, domains of engagement throughout the course of these Canadian patients being treated while abroad.

Enabled by the size of the dataset, thematic coding was conducted by hand in a word processing program with organizational and analytic codes being identified and confirmed by the team. After hand coding was complete, the lead investigator shared coded extracts for each of the three environments to seek confirmation from the team regarding their interpretation, including their scope and scale. In the section that follows we include extracts in the form of verbatim quotes in order to enable the participants to ‘speak’ to the issues at hand. The inclusion of these quotes, in addition to our use of investigator triangulation at multiple points and establishment of an audit trail by keeping a record of important decisions, contribute to the rigour of this analysis [31, 32].

## Results

A total of 27 people participated in the three focus groups, 12 nurses, six physicians, and nine administrators. Participants, almost 75% (*n* = 20) of whom were women, had worked in the hospital for an average of 18.6 years. All had deep experience treating, overseeing the care of, and/or developing administrative protocols for older Canadian patients. Participants indicated there were no seemingly common treatments accessed by this transnational patient group, whether as in- or out-patients. Instead, it was noted Canadians presented for both chronic and acute care in most hospital units. It was also explained that in some cases Canadians called well ahead of their arrival in Yuma to schedule treatments to facilitate care continuity (e.g., diabetes management, cancer therapies). Participants also widely agreed it was very common to see admissions of Canadians through emergency in the winter months. These admissions, in some cases, were due to pharmaceutical interactions with sun, heat, and/or alcohol. It was recognized Canadian retirement migrants, as well as retirement migrants coming from elsewhere in the US, were an important patient group for the hospital. In fact, the participants shared how the facility increased staff, opened extra beds, and provided additional services (including some that are community-based) during the winter months when the population of Yuma almost doubled with the influx of older retirees.

We asked participants to comment on the practical realities of treating older Canadian retirement migrants, many of which were framed as either opportunities or challenges for the hospital, clinicians, wider community, and/or patients. Through the process of thematic analysis it became clear that distinct opportunities and challenges emerged in different types of environments encountered in the care trajectory. In the remainder of this section we explore three specific environments: the practice environment, the transnational environment, and the community environment. By ‘practice environment’ we are referring to the spaces and activities that centre on caring for older Canadians within the hospital, including facilitating care continuity. The transnational environment pertains to the administrative, information exchange, and other processes that necessitate involvement of health care and insurance providers and spaces at home in Canada, and abroad in Yuma. Regarding the community environment, we conceptualize it to be the larger setting within which the hospital is located that shapes the everyday lives of both patients and those working at this hospital.

### Practice environment

Within the practice environment, the opportunities for treating a sizeable Canadian retirement migrant patient load focus primarily on health human resources staffing and training. For example, participants noted that the presence of these patients assists with creating and maintaining a vibrant working environment as they keep staff busy with people in need of care. This demand facilitates staff retention among those with an interest in geriatric medicine, serves as the basis for placing medical residents at the hospital, and necessitates bringing in seasonal health human resources that expand treatment opportunities. The need to hire seasonal staff on a yearly basis has led to the development of a strong pool of returning seasonal health human resources. *“I would say at least 70 to 75% of our seasonal employees have been coming back for at least five years, if not more…they’re up to date on every change that we’ve done*.” Participants referred to returning health workers’ familiarity with treating Canadian international retirement migrants as a benefit of having seasonal employees, in that they are well prepared to understand the nuances of dealing with this mobile patient group (e.g., knowing to ask about both local and Canadian care networks upon admission). Having returning workers also lessened the seasonal training and orientation burden on administrators and other hospital staff, which was beneficial as such resources could be directed elsewhere.

The seasonal influx of older Canadian patients into the practice environment brings with it billing and practical complexities that pose challenges. A significant one is the need to navigate Canadian travel medicine insurance policies. Participants reported stress both for hospital staff and Canadian patients while waiting for insurance approvals, some of which needed to come from Canadian insurers that insurance and billing staff were unfamiliar with. Another complexity pertains to the lack of continuity of care for these Canadian patients who return home at the end of the travel season. This negatively affects the abilities of all staff, whether administrative or clinical, to provide necessary follow-up. At the same time, this has prompted some health workers to identify opportunities for enhancing continuity in this particular transnational care context. For example, some participants spoke about calling Canadian patients’ regular care providers directly as a strategy for enhancing informational continuity, though this was often difficult due to time zone and administrative differences. An interesting administrative challenge in the clinical environment is that it is not always easy to determine who is a Canadian patient. The reason for this is that many Canadians have permanent or long-term residences where they stay in Yuma, and upon admission to the hospital they often report these local addresses. *“And I think that we struggle at times because of how they provide information, unit number or lot number or, they might consider [this as] their year-round residency, but, you know, in the summertime they go somewhere else. I struggle just getting correct addresses.”* This was cited as a challenge because it may delay reaching out to international travel health insurers.

### Transnational environment

The transnational environment provides opportunities to enhance communication networks between international retirement migrants, physicians in Yuma, and clinicians based in Canada. This includes some physicians taking the time to call care providers at home in order to discuss post-discharge treatment protocols and understand opportunities for continued care at home in a health system that participants were mostly unfamiliar with. In instances where patients were managing cancer transnationally: *“So, communication from Canada to us…oncology is a lot more open to people moving, because we’ll get people [who] come down with ports and we’ll get orders to do lab work every month while they’re here.”* Participants specifically cited the hospital’s community outreach programs as being crucial to improving communication networks and mitigating issues pertaining to the lack of continuity of care experienced by Canadians and other seasonal residents. For example, a free screening program has attracted a sizeable number of Canadian patients. *“Our database shows a little over 6,000 active members within the program…and out of that almost 2,000 of our members are Canadians.”* Participants indicated the outreach programs are not only utilised to help educate the local population, but also act as an effective means to introduce older Canadians to local health care services, address concerns they have about their lack of familiarity with US-based health care, and to register in the hospital’s database *prior* to requiring admission. Participants also described efforts to proactively educate Canadian international retirement migrants about the importance of traveling abroad with medical records in these outreach efforts.

The transnational environment creates significant administrative challenges for health professionals. These challenges are related to interactions with international health insurance providers and the lack of familiarity with the home health care systems of older Canadian patients. Participants expressed frustration over the time spent waiting for insurance provider clearance and described it as a significant source of stress for both patients and health workers in a context where many older Canadians are already concerned about their abilities to return home or afford care in the US. *“You get frustrated because your hands are tied. You’ve got to wait before you can actually give them the care that they need.”* This frustration can become amplified: *“And they’ll [Canadian patient] have like, the note when you open up the chart. It’ll be like right, you know, in one of the main places where you see, it’ll say ‘need pre-authorization for whatever, call if you have questions’.”* There was also widespread acknowledgement that hospital workers’ lack of familiarity with Canadian health care systems and Canadian patients’ opportunities for care at home added complexity to the transnational environment. This lack of familiarity left some physicians and administrators unclear as to how to best advocate for their Canadian patients when interacting with international travel health insurance providers (e.g., in justifying why returning home or treatment in Yuma was best). It was also reported that no standard process exists for informing providers in Yuma about patient outcomes once they had returned to Canada. *“What happens to the patients when they go back, then? Are they treated exactly as they would be treated here, or what?”* This lack of ‘closure’ left some health workers with anxiety and stress related to the uncertainty associated with regularly treating transnational patients.

### Community environment

The increased seasonal older population in Yuma provides opportunities for the hospital and its staff within the wider community environment by bolstering the number of hospital volunteers, facilitating the development of informal care networks, as well as creating and spreading a culture of care. As one participant explained, Canadian retirement migrants *“get a sense of community, at least with the hospital. So, there’s a lot of them participating [volunteering] and they actually feel that they’re part of…this community. So, I think that’s really helpful*.” These Canadian volunteers assist with support and navigation, providing information and in-hospital transportation, among other things, and participants explained they are a vital part of the network that connects the hospital to the community. The ‘retirement migrant culture’ that some older Canadians are part of in Yuma extends to the development of informal care networks that support patients within and beyond the hospital. “*Yeah, the culture is ... they take good care of each other, they’re a good support group for each other.*” These informal care networks lessen the burden on the hospital staff in that they ease transitions back into the community. *“You can see it in the hospital…the snowbirds visit the snowbirds. I mean they have a really tight culture, and they’ve been seeing the same people from all over the country and Canada and they all meet at the same park every year...”* It was explained that while older Canadians create strong informal care infrastructure and support for one another, this is particularly true for those who live in the same residential parks.

The community environment presents some challenges for the hospital and its staff associated with lifestyle factors observed among some Canadian international retirement migrants. For example, it was widely discussed that the party atmosphere that surrounds international retirement migration results in significant numbers of injuries due to alcohol consumption. *“They [snowbirds] have fun. They trip and fall, end up in radiology again, yeah. The margaritas…the margaritas do it every time!”* Physicians also discussed a general lack of education about safe sex among older patients and how this has resulted in growing rates of sexually transmitted infections being reported. Canadian retirees also undertake other high-risk activities that can cause further stress on the hospital and its staff. For example, Canadian snowbirds routinely travel from Yuma to Los Algodones, Mexico (a short trip by car or bus) to purchase affordable dental care, pharmaceuticals, and other treatments. *“The ones that go to…Mexico to get…alternative treatments that you cannot get here. And then you’re like, ‘don’t do it!’ And you see them in the ER [emergency room] or in the hospital when they have an exacerbation of whatever.”* Complications resulting from these community-based practices place significant pressure on hospital staffing plans during the busy winter months, thereby posing as a challenge for the hospital that emerges from the community environment.

## Discussion

Despite some long standing and highly cited existing research that suggests Canadian retirement migrants have little uptake of health care while in the US (e.g. [9, 15, 33, 34]), this analysis has shown that in Yuma, Arizona Canadians are indeed a significant patient group. In fact, many focus group participants viewed older Canadian patients to be a growing revenue stream. By consulting directly with health care providers and administrators at the only hospital in this city and the surrounding region, our results summarized in Table 1 show particular opportunities and challenges emerge in specific care environments when treating or connecting with this transnational patient population. Important opportunities include the creation of a strong and diverse seasonal workforce in the hospital, new transnational paths of communication and information sharing for physicians and health administrators, and informal care networks that support formal health care services within and beyond the hospital. These opportunities are balanced out by billing, practical, administrative, and lifestyle-related challenges which add complexity to treating this group of transnational patients. We characterized the opportunities and challenges that exist for the hospital, physicians, patients, larger community and other groups across three distinct environments of care: the practice environment, the transnational environment, and the community environment. Although we discussed these environments separately in the results section, it is important to acknowledge they are interrelated and opportunities or challenges that emerge in one may be seen another way in a different environment. We begin this section by exploring some of these intersections, after which we identify some pressing new research directions.

**Table 1:** Synthesis of the Opportunities and Challenges in Providing Health Care to Canadian International Retirement Migrants in Yuma, Arizona

	*Scope of this Environment*	*Example of a Challenge Created in this Environment*	*Example of an Opportunity Created in this Environment*
*Practice* *Environment*	The spaces and activities that centre on caring for Canadian international retirement migrants within the hospital	Providing needed follow-up care and facilitating care continuity for Canadian patients who will return home at the end of the season	Hiring and retaining hospital staff to meet the seasonal influx in demand for services
*Transnational* *Environment*	The administrative, information exchange, and other processes that involve both health care or travel health insurance providers at home (in Canada) and abroad (in Yuma)	Navigating lack of familiarity with Canadian health care system and Canadian patients’ health care opportunities at home	Educating Canadian international retirement migrants about the importance of travelling abroad with medical records
*Community* *Environment*	The larger setting within which the hospital is located that shapes the everyday lives of patients and hospital workers	Caring for Canadian retirement migrant patients who have undertaken risky activities associated with ‘party atmosphere’ in snowbird communities	Creating a culture of care that extends beyond the hospital and into the community (e.g., building a hospital volunteer program that includes Canadians)

The opportunities and challenges identified in this thematic analysis intersect across the three environments of care explored. One such intersection lies with the billing, practical, and administrative challenges identified within the practice and transnational environments. Participants’ lack of knowledge of the Canadian health care system and lack of established relationships with some Canadian travel health insurance providers underlie these distinct challenges. For example, participants in all focus groups spoke openly about their confusion with the Canadian health care system, which was identified as a major source of stress associated with treating Canadian patients. This lack of knowledge created several challenges in the practice and transnational environments. It was not uncommon for participants to discuss frustration with their perception that the ‘Canadian health care system’ can require a patient to return home for care; meanwhile, it is actually private travel health insurance providers that make the decisions participants spoke of and *not* the ‘Canadian health care system’ they implicated. Consistent with our study, existing research highlights the complexity of crossing health systems and the challenges this can pose while abroad. For example, there is a long-standing and sizeable body of research that explores the cross-cultural complexity emerging from accessing health care transnationally [35–39]. Given the cultural familiarity between Canada and the US [36, 37], it is not surprising that this well-established challenge did not emerge as an important discussion point among participants. However, other research has shown how a lack of familiarity with patients’ home health care systems can generate misunderstandings and other challenges in the context of transnational care. For example, research on medical tourism has shown that patients may not understand differences in the roles that health workers in destination hospitals take on relative to what they are familiar with in their home systems, which can create confusion [40, 41]. Further to this, in the context of caring for ill and injured vacationers, interviews with health care providers in a popular vacation destination in Mexico point to how misunderstandings based on stereotypes regarding practice competency can affect patients’ decisions regarding receiving treatment for injuries sustained on holiday while abroad [42].

The continuity and dependability of the ‘snowbird season’ provides overlapping opportunities in the practice and community environments, which serves as the basis for another important intersection in our thematic findings. Because the seasonal population influx in Yuma can be relied upon, although specific numbers may vary annually, the hospital was able to meaningfully expand its seasonal operations to include additional health workers and community outreach activities that benefitted all retirement migrants, including those from Canada. While a number of existing studies have touted the economic benefits to local communities that host Canadian retirees who winter in the US [9, 15, 33, 34], the current analysis provides unique insight into how the operations of a specific hospital benefits from this transnational mobility. These benefits result in creating opportunities in the practice and community environments that form the basis of the transnational care received by older Canadians in Yuma, which included community outreach initiatives with significant uptake. These benefits also facilitate the development of distinct types of continuity. For example, the clinicians, nurses and physicians operating in the practice environment benefitted from the return of some of the same seasonal health professionals yearly, while the community environment provided experienced retirement migrant volunteers for the hospital. According to participants, the combination of these two opportunities created a positive working environment and enhanced the work culture of the hospital during snowbird season. Existing research shows this advantage exists in other industries that rely on seasonal workers, such as the tourism sector. Similar references to opportunities associated with developing a sense of community that positively integrates seasonal workers emerge from the tourism sector, as do those pertaining to the additional opportunities generated by cost savings for the employers of hiring seasonal workers [43, 44].

Interestingly, participants characterized the community environment in both positive and negative ways. For example, it was explained that some Canadian retirement migrants chose to become volunteers for the hospital, who create micro-communities of care within this facility and assist Canadian patients and others with transitioning back into the community after discharge. These individuals were highly-valued and their presence created important opportunities both for the hospital and its patients. Their presence in the hospital was also thought to reflect the tight-knit nature of retiree communities and the strong and positive social focus of the international retirement migration lifestyle (e.g. [45]). Meanwhile, participants in all focus groups identified the wider community to be one that poses some lifestyle-associated health risks for older Canadians. The intersection of alcohol overconsumption, risky sexual practices, and/or opting to purchase dental care or pharmaceuticals in Mexico with the increased health risks associated with aging resulted in some Canadian patients placing particular demands on the hospital and health workers. While we acknowledge the participants have interacted with only a fraction of the Canadian retirees who winter in Yuma, their characterization of the health risks associated with the community environment counter wide-held understandings of older people leading quiet, safe, risk-adverse lives [46, 47]. These findings provide important insight into *why* some Canadian retirement migrants need to access health care while in the US and highlights the importance of the wider outreach activities the case study hospital undertakes with all retirement migrants in the community environment.

To the best of our knowledge, this analysis provides the first insights offered by health care providers and administrators of the challenges and opportunities associated with treating international retirement migrants in the hospital setting. It is thus not surprising that many directions for future research build from the results, and here we highlight four. First, very little is known about if and how international retirement migrants’ home health care providers are affected by this transnational practice. What are their experiences of supporting these patients before they go abroad and after they have returned? Have they interacted with health workers in the destination for any reason? Do they share the concerns about care and informational continuity raised by the focus group participants in this study? Exploring such questions has the potential to deepen the insights gleaned in the current study. Second, given the sizeable number of Canadians who participate in international retirement migration and the country’s rapidly aging population [42], it is an opportune time to explore models and mechanisms for enhancing care continuity for those who participate in this transnational practice. Doing so has the potential to respond to some of the challenges identified by the focus group participants. Are there ways to facilitate information sharing and collaboration between health care providers/facilities at home and abroad that are cost-effective and maintain privacy? What are the potential risks and benefits associated with doing so, and what degree of involvement should private travel health firms have? Capturing reliable demographic data about Canadian international retirement migrants who use health care while abroad will also assist with enhancing these analyses and targeting interventions that support continuity of care. Third, conducting similar research with health care providers and administrators in other international retirement migrant destinations and with different transnational patient populations will assist in identifying which findings from the current analysis are transferrable to other contexts. Finally, while we noted references of visits from friends and family to older Canadians in the hospital, the current study was focused on formal caregiving and did not explore informal caregiving in the international retirement migration context. Do unpaid, informal caregivers such as friends and family members encounter similar types of opportunities and challenges to those cited by the formal caregivers consulted in this study? Can informal caregivers play a role in mitigating or eliminating some of the challenges documented in this analysis and enhancing some of the opportunities? These are questions worth of exploration given the significant role that informal caregivers play in managing health and wellbeing among older populations [48–51].

## Conclusion

In this exploratory qualitative analysis, we presented the findings of focus groups conducted with health care providers and administrators working at a regional hospital in Yuma, Arizona regarding their experiences of treating older Canadian retirement migrants who winter in this destination. Through thematic analysis we identified specific opportunities and challenges posed by treating this transnational patient population that transect three distinct environments encountered in the hospital-based care trajectory: the practice environment, the transnational environment, and the community environment. The findings point to challenges associated with obtaining approvals for care from travel health insurance providers, lack of familiarity with Canadian patients’ home health care systems, informational continuity, and the risky behaviours associated with the international retirement migration lifestyle that bring some patients to the hospital. There are, however, opportunities associated with this transnational population including seasonal increases in staffing, community outreach by the hospital, the creation of new forms of communication, and an influx of Canadian hospital volunteers during ‘snowbird season’ who can assist with reintegration into the community after discharge. While additional research is needed in order to deepen our understanding of these findings and their implications, the experiences reported in this study work to counter the notion that older Canadians who stay seasonally in the US have little interaction with local health systems while abroad.

## Data Availability

The focus group data are not publicly available.

## Abbreviations

ER: Emergency room
US: United States of America

## References

[1] Rodriguez V, Fernández-Mayoralas G, Rojo F (2004). International retirement migration: retired Europeans living on the Costa del Sol, Spain. Popul Rev.

[2] King R, Warnes T, Williams A (2000). Sunset lives: British retirement migration to the Mediterranean.

[3] O'Reilly K (2000). The new Europe/old boundaries: British migrants in Spain. J Soc Welfare Fam Law.

[4] Coates KS, Healy R, Morrison WR (2002). Tracking the snowbirds: seasonal migration from Canada to the USA and Mexico. Am Rev Can Stud.

[5] Desrosiers-Lauzon G (2009). Canadian snowbirds as migrants. Canadian Issues.

[6] Jerkovic J, Kealey M. Report on the Feasibility of Cross-Border Healthcare for Canadians in Surprise. Arizona; 2018. Available from: https://www.surpriseaz.gov/DocumentCenter/View/39743/2018-Cross-Border-Healthcare-Feasibility-Report?bidId.

[7] Trade commissioner, Government of Canada (2018). Canada-Florida economic impact study: Canada is Florida’s most important economic partner. N.d.

[8] Tasker JP (2017). Snowbirds could soon stay 8 months in U.S. despite push to tighten border | CBC News.

[9] Longino CF, Marshall VW, Mullins LC, Tucker RD (1991). On the nesting of snowbirds: a question about seasonal and permanent migrants. J Appl Gerontol.

[10] Hall K, Hardill I (2016). Retirement migration, the ‘other’ story: caring for frail elderly British citizens in Spain. Ageing Soc.

[11] Northcott HC, Petruik CR (2011). The geographic mobility of elderly Canadians. Can J Aging/La Revue canadienne du vieillissement.

[12] Vogeli C, Shields AE, Lee TA, Gibson TB, Marder WD, Weiss KB, Blumenthal D (2007). Multiple chronic conditions: prevalence, health consequences, and implications for quality, care management, and costs. J Gen Intern Med.

[13] Ward BW, Schiller JS, Goodman RA (2014). Multiple chronic conditions among us adults: a 2012 update. Prev Chronic Dis.

[14] Wolff JL, Starfield B, Anderson G (2002). Prevalence, expenditures, and complications of multiple chronic conditions in the elderly. Arch Intern Med.

[15] Marshall VW, Longino CF, Tucker R, Mullins L (1989). Health care utilization of Canadian snowbirds: an example of strategic planning. J Aging Health.

[16] Dwyer PJ (2000). Movements to some purpose? An exploration of international retirement migration in the European Union. Educ Ageing.

[17] Ackers L, Dwyer P (2004). Fixed laws, fluid lives: the citizenship status of post-retirement migrants in the European Union. Ageing Soc.

[18] Innes A (2008). Growing older in Malta: experiences of British retirees. Int J Ageing Later Life.

[19] Croucher S (2012). Privileged mobility in an age of globality. Societies..

[20] Howard RW (2008). Western retirees in Thailand: motives, experiences, wellbeing, assimilation and future needs. Ageing Soc.

[21] Ontario Health Insurance Program (2017). OHIP coverage while outside Canada.

[22] Then KL, Rankin JA, Ali E. Focus group research: what is it and how can it be used?. Can J Cardiovasc Nurs. 2014;24(1). 24660275

[23] Morgan DL (1996). Focus groups. Annu Rev Sociol.

[24] Breen RL (2006). A practical guide to focus-group research. J Geogr High Educ.

[25] US Census Bureau (2018). Yuma county quick facts report.

[26] Flyvbjerg B (2006). Five misunderstandings about case-study research. Qual Inq.

[27] Yin RK. Case study research and applications: design and methods. Los Angeles: Sage publications; 2017.

[28] Saunders B, Kitzinger J, Kitzinger C (2015). Anonymising interview data: challenges and compromise in practice. Qual Res.

[29] Braun V, Clarke V, Hayfield N, Terry G (2018). Thematic analysis. Chapter 4. Handbook of research methods in health social sciences.

[30] Javadi M, Zarea K (2016). Understanding thematic analysis and its pitfall. J Client Care.

[31] Mays N, Pope C (1995). Qualitative research: rigour and qualitative research. Bmj..

[32] Seale C, Silverman D (1990). Ensuring rigour in qualitative research. The European journal of public health. 1997 Dec 1;7(4):379-84Longino CF, Marshall VW. North American research on seasonal migration. Ageing Soc.

[33] Longino CF, Taplin IM (1994). How does the mobility of the elderly affect health care delivery in the USA?. Aging Clin Exp Res.

[34] Betancourt J, Green A, Carillo JE (2000). The challenges of cross-cultural healthcare-diversity, ethics, and the medical encounter. Bioethics Forum.

[35] Hofstede G. Culture’s consequences: International differences in work-related values. Beverly Hills: Sage; 1980.

[36] Selmer J (2007). Which is easier, adjusting to a similar or to a dissimilar culture? American business expatriates in Canada and Germany. Int J Cross-cult Manag.

[37] Suphanchaimat R, Kantamaturapoj K, Putthasri W, Prakongsai P (2015). Challenges in the provision of healthcare services for migrants: a systematic review through providers’ lens. BMC Health Serv Res.

[38] Hennebry J, McLaughlin J, Preibisch K (2016). Out of the loop: (in) access to health care for migrant workers in Canada. J Int Migr Integr.

[39] Wang L, Kwak MJ (2015). Immigration, barriers to healthcare and transnational ties: a case study of south Korean immigrants in Toronto, Canada. Soc Sci Med.

[40] Solomon H (2011). Affective journeys: the emotional structuring of medical tourism in India. Anthropol Med.

[41] Crooks VA, Casey V, Whitmore R, Johnston R, Snyder J, Lunt N, Hanefeld J, Horsfall D (2015). ‘They go the extra mile, the extra ten miles…’: examining Canadian medical tourists’ interactions with health care workers abroad. Elgar handbook on medical tourism and patient mobility.

[42] Hoffman L, Crooks VA, Snyder J (2018). A challenging entanglement: health care providers’ perspectives on caring for ill and injured tourists on Cozumel Island, Mexico. Int J Qual Stud Health Well-being.

[43] McCole D (2015). Seasonal employees: the link between sense of community and retention. J Travel Res.

[44] Anderson E (2018). Belonging, temporariness and seasonal labour: working holidaymakers’ experiences in regional Australia. Chapter 10. Work and identity.

[45] Campbell N (2015). Designing for social needs to support aging in place within continuing care retirement communities. J Housing Built Environ.

[46] Albert SM, Duffy J (2012). Differences in risk aversion between young and older adults. Neurosci Neuroecon.

[47] Rolison JJ, Hanoch Y, Wood S, Liu PJ (2013). Risk-taking differences across the adult life span: a question of age and domain. J Gerontol B Psychol Sci Soc Sci.

[48] Lee R, Mason A (2014). Is low fertility really a problem? Population aging, dependency, and consumption. Science.

[49] Naganathan G, Kuluski K, Gill A, Jaakkimainen L, Upshur R, Wodchis WP (2016). Perceived value of support for older adults coping with multi-morbidity: patient, informal care-giver and family physician perspectives. Ageing Soc.

[50] van Groenou MI, De Boer A (2016). Providing informal care in a changing society. Eur J Ageing.

[51] Van Houtven CH, Norton EC (2004). Informal care and health care use of older adults. J Health Econ.

